# CRISPR/Cas9-Correctable mutation-related molecular and physiological phenotypes in iPSC-derived Alzheimer’s *PSEN2*^*N141I*^ neurons

**DOI:** 10.1186/s40478-017-0475-z

**Published:** 2017-10-27

**Authors:** Maitane Ortiz-Virumbrales, Cesar L. Moreno, Ilya Kruglikov, Paula Marazuela, Andrew Sproul, Samson Jacob, Matthew Zimmer, Daniel Paull, Bin Zhang, Eric E. Schadt, Michelle E. Ehrlich, Rudolph E. Tanzi, Ottavio Arancio, Scott Noggle, Sam Gandy

**Affiliations:** 1 Department of Neurology, NFL Neurological Care Center, Icahn School of Medicine at Mount Sinai, New York, NY 10029 USA; 2 Department of Psychiatry, Alzheimer’s Disease Research Center, Icahn School of Medicine at Mount Sinai, New York, NY 10029 USA; 3 The New York Stem Cell Foundation Research Institute, New York, NY USA; 4 Universidad Francisco de Vitoria, 28223 Pozuelo de Alarcón, Madrid, Spain; 5 Present Address: Taub Institute and the Department of Pathology and Cell Biology, Columbia University, New York, NY 10032 USA; 6 Department of Genetics and Genomic Sciences, Icahn Institute for Multi-Scale Biology, Icahn School of Medicine at Mount Sinai, New York, NY 10029 USA; 7 Department of Pediatrics, Icahn School of Medicine at Mount Sinai, New York, NY 10029 USA; 8 Genetics and Aging Unit, Department of Neurology, Massachusetts General Hospital, Boston, MA 02114 USA; 9 Department of Pathology and Cell Biology, Columbia University, New York, NY 10032 USA

**Keywords:** Alzheimer’s disease, iPSC, BFCN, CRISPR/Cas9, Electrophysiology, Basal forebrain, Cholinergic, Presenilin, PSEN2

## Abstract

Basal forebrain cholinergic neurons (BFCNs) are believed to be one of the first cell types to be affected in all forms of AD, and their dysfunction is clinically correlated with impaired short-term memory formation and retrieval. We present an optimized in vitro protocol to generate human BFCNs from iPSCs, using cell lines from presenilin 2 (*PSEN2)* mutation carriers and controls. As expected, cell lines harboring the *PSEN2* ^*N141I*^ mutation displayed an increase in the Aβ42/40 in iPSC-derived BFCNs. Neurons derived from *PSEN2* ^*N141I*^ lines generated fewer maximum number of spikes in response to a square depolarizing current injection. The height of the first action potential at rheobase current injection was also significantly decreased in *PSEN2* ^*N141I*^ BFCNs. CRISPR/Cas9 correction of the *PSEN2* point mutation abolished the electrophysiological deficit, restoring both the maximal number of spikes and spike height to the levels recorded in controls*.* Increased Aβ42/40 was also normalized following CRISPR/Cas-mediated correction of the *PSEN2* ^*N141I*^ mutation. The genome editing data confirms the robust consistency of mutation-related changes in Aβ42/40 ratio while also showing a *PSEN2*-mutation-related alteration in electrophysiology.

**Electronic supplementary material:**

The online version of this article doi: (10.1186/s40478-017-0475-z) contains supplementary material, which is available to authorized users.

## Introduction

The “amyloid hypothesis” is one of the most popular formulations for the pathogenesis of Alzheimer’s disease (AD). Recent examples of clinicopathological and/or clinicoradiological dissociation have led to the consideration of alternative models in order to explain, respectively, why neuropathological AD is not always associated with dementia [24], and why about one-third of patients with clinical AD have negative amyloid brain scans [40]. It has been proposed that clinical AD can be caused by one of several “feed-forward” scenarios linking amyloidosis, tauopathy, neuroinflammation, and neurodegeneration [22]. Mutations in the gene encoding presenilin 2 (*PSEN2*) are associated with autosomal dominant early onset familial Alzheimer’s disease (EOFAD). The linkage of a locus on human chromosome 1q31–42 linked to EOFAD led to the identification of the *PSEN2* ^N141I^ point mutation in the Volga German kindreds in 1995 [43]. This mutation causes elevation in the Aβ42–43/40 ratio, thereby promoting assembly of Aβ oligomers and fibrils [83].

In considering the progression of AD, human basal forebrain cholinergic neurons (BFCNs) are one of the first cell types whose dysfunction underlies the early loss of short-term memory recall in all forms of AD. The “cholinergic hypothesis of AD” was formulated in the mid-1970s [6, 20, 61], and the discoveries of reduced acetylcholine release from neurons of the nucleus basalis of Meynert confirmed the presence of a presynaptic cholinergic deficit in the basal forebrain of AD patients [1, 71]. Based on those observations, acetylcholinesterase inhibitors were developed and continue as the most widely used symptomatic treatments for AD [21, 28, 33, 82]. Eventually, post-mortem brain biochemical and volumetric studies at different stages of the disease identified several other regions of the brain that were also affected early in the course of AD [63]. These studies have traditionally focused on the hippocampus and cortex, but more recently, attention has begun shifting back to the basal forebrain and adding other areas, such as the striatum [27, 62]. The latest analyses suggest that cholinergic basal forebrain volume measurement may be a better predictor of the transition from MCI to AD than the previous standard, hippocampal volume [10].

We previously reported the generation of iPSC-derived neurons from banked fibroblasts from subjects harboring *PSEN1* ^*A246E*^ and *PSEN1* ^M146L^ mutations [77]. In characterizing the gene expression profiles from these iPSC-derived neurons, we observed an unexpected association of elevated expression of the inflammasome gene *NLRP2* in undifferentiated *PSEN1* mutant iPSCs and their and neuronally differentiated progeny [77]. This led us to examine NLRP2 expression in our *PSEN2* mutant lines and employ CRISPR/Cas9 [15] to investigate if activation of the inflammasome was tightly linked to the pathogenic mutation in *PSEN2*. While we did not find altered expression of NLRP2 in gene-corrected *PSEN2* lines, we observed significant mutation-related, editing-reversible differences in excitability of BFCNs.

## Materials and methods

### Generation and maintenance of iPSC lines

7889(s)B, 050643 (Control), 948 (AD1), 949(fControl), and 950 (AD2) iPSC lines were obtained via the NYSCF Repository following the guidelines from [60]. The derivation and characterization of Nkx2.1-GFP ESC line was previously published [30]. ES and iPS cell lines were expanded and maintained in serum-free mTeSR1 media (Stem Cell Technologies). Cells were lifted using StremPro Accutase (ThermoFisher) and media was supplemented with 10 μM ROCK inhibitor (Y27632, Stemgent) during cell passaging.

For all studies in this paper, cell lines underwent at least 3 independent differentiations from the iPSC stage to the mature neuron stage. Data were routinely compared across these independently derived genotype-identical neurons (or in some cases neuronal precursors), and if comparable results were obtained across independently genotype-identical derived cells, they were considered to be qualified representatives of their genotype and so were passed along for genotype-specific experimentation.

### Aβ42 oligomer preparation

Aβ42 oligomers were prepared as previously reported [23, 78]. Briefly, we dissolved 1 mg of Aβ42 (American Peptide Company) in 1,1,1,3,3,3-hexafluoro-2-propanol (HFIP) (Sigma). This preparation was aliquoted and dried using a SpeedVac centrifuge. The pellet was then resuspended in DMSO to obtain a 5 mM solution which was sonicated in a water bath for 10 min. From here aliquots were stored at -20C and used within 2 weeks by diluting with 100 μl of PBS and leaving for 12 h at 4 °C in order for oligomerization to proceed. This final solution was diluted 1:16 in cell media for studies, allowing cells to be exposed to 5 μM of Aβ42 oligomers. Control wells were diluted with 1:16 PBS. Cells were exposed to oligomers or PBS without media change for a period of 3 days.

### Cell death assays

Cells were assayed in a 96-well plate format. Oligomer or vehicle solutions were added to media and allowed to incubate for a period of 3 days. Media was then collected and assayed using a lactate dehydrogenase toxicity assay (Thermo Fisher Scientific). 50 μl of media and an equal amount of reaction mix buffer were incubated for a period of 30 min. An additional set of wells per experiment were treated with 2% Triton X-100 for a 5-min period in order to lyse all cells, and media from these wells was also collected and incubated as described. After incubation absorbance was recorded at 490 nm and 680 nm, signal and background absorbance, respectively. Signal values were subtracted from background, and values were adapted to the total LDH content as determined by Triton X-100 treated wells. Propidium iodide (Thermo Fisher Scientific) was added to cell media for a 1 μM final concentration and allowed to incubate for 5 min. Cells were then washed twice with media and imaged. Images were captured using CELIGO image cytometer and accompanying software (Nexcelom Bioscience). Each biological variable was assessed in technical triplicates within each designated “Experiment”, and each designated “Experiment” was performed in at least three complete “start to finish” iterations.

### Differentiation of basal forebrain cholinergic neurons from iPS and ES cells

Human ES or iPSC were plated as single cells after chemical dissociation using Accutase (Sigma-Aldrich) into Cultrex (Trevigen) coated plates, at a density of 4–8 × 10^5^ cells per well in 6-well plates or petri dishes and adapting cell numbers. Cells were initially maintained in mTeSR1 media (Stem Cell Technologies) until reaching full confluency. On “day 0” of differentiation, media was replaced by Custom mTeSR1 media (Stem Cell Technologies) lacking factors promoting pluripotency i.e., bFGF, TGF-Beta, Li-Cl, GABA and pipecolic acid. The addition of dual SMAD inhibitors (SB431542 10 μM plus LDN193189 250 nM, Selleckchem) at day 0 drives cells towards neuroectoderm specification. At day 2 of differentiation, media was replaced by Custom mTeSR1 containing dual SMAD inhibitors plus two ventralizing agents: SAG at 500 nM (R&D) and Purmorphamine at 2 μM (Stemgent). Cells were fed every 2 days with this media until day 9, when media was progressively switched to Brainphys media (Stemcell Technologies) supplemented with B27 (Life Technologies) [3]. Neural progenitors were harvested at day 11 using Accutase, p75^+^ (CD271) NPCs were purified by FACS and plated at a density of 80,000 cells per well into non-adherent 96 well V-bottom plates in Brainphys + B27 supplemented with 10 μM ROCK inhibitor (Y27632, Stemgent), Nerve Growth Factor, NGF, (Alamone labs, 50 ng/mL) and Brain Derived Neurotrophic Factor, BDNF, (R&D, 50 ng/mL). Cells were allowed to aggregate and form Neuronal Embryoid Bodies (NEBs) and were fed every other day until day 19. At day 19 NEBs were dissociated using Accutase (Sigma-Aldrich) and were plated as monolayer cultures on plates coated with branched polyethynilimine (.1%, Sigma-Aldrich) and laminin (10 mg/mL, Life Technology) in Brainphys media + B27 supplement with BDNF and NGF. The media was changed every 2 days until analysis. As an alternative, 3D NEBs were dissected manually into 3–4 pieces for expansion and further grown, or were cryopreserved. Initial versions of the protocol used Neurobasal as a base media instead of BrainPhys.

### Genomic DNA isolation and sequencing

Genomic DNA was isolated from PSEN2 mutant or control iPSC lines using High Pure PCR Template Preparation Kit (Roche) following manufacturer instructions. Genomic samples were treated with RNAse (QIAGEN) prior to amplification. A fragment from exon 5 of PSEN2 containing PSEN2^N141I^ mutation was amplified using the following primers: Forward 5′-CATCAGCCCTTTGCCTTCT-3′, Reverse: 5′-CTCACCTTGTAGCAGCGGTA-3′, generating a 173 bp fragment, regardless of the genotype. For detection of ApoE allelic variants, a fragment of 244 bp was amplified prior to sequencing using the primers: Forward: 5′-ACAGAATTCGCCCCGGCCTGGTACAC-3′, Reverse: 5′-TAAGCTTGGCACGGCTGTCCAAGGA-3′. Both PCR were performed with the following settings: 10 min 94C, 40 cycles (30 s 94C, 20s 62C, 10s 72C) 7 min 72C. PCR products were run in a 2% agarose gel to check the size of the amplified fragment. After amplification, samples were cleaned using EXOSAP-it (Thermo Fisher Scientific) and then sequenced using the following primers: PSEN2 (Forward: 5′-TCAGCATCTACACGCCATTC-3′, Reverse: 5′-AGCACCACCAAGAAGATGGT-3′), from [53]; ApoE (Forward: 5′- ATTCGCCCCGGCCTGGTACACTGCCA-3′, Reverse: 5′- CTGTCCAAGGAGCTGCAGGCGGCGCAG-3′), from [36].

$$ \mathbf{Bold}\ \mathrm{highlight}=\mathrm{base}\  \mathrm{of}\  \mathrm{the}\  \mathrm{ssODN}\  \mathrm{that}\  \mathrm{corrects}\  \mathrm{the}\  \mathrm{point}\  \mathrm{mutation} $$

### CRISPR/Cas9 gene correction

iAD1 Control and iAD2 Control lines were originated from 948 (AD1) and 950 (AD2) iPSC lines by CRISPR/Cas9-mediated correction of the *PSEN2* ^*N141I/WT*^ heterozygous point mutation to *PSEN2* ^*WT/WT*^. g1N141I single guide RNA (sgRNA) was cloned into pSpCas9(BB)-2A–GFP (PX458) vector, generating pSpCas9-g1N141I-GFP vector to direct gene editing to the sequence in exon 5 of PSEN2 where the Volga mutation is located. Single stranded oligonucleotides (ssODN) are efficient templates for the CRISPR/Cas9 correction [13, 66]. ssODN#A-N141I (sequence detail below) was used as donor sequence for gene correction. We designed asymmetric ssODN sequences with a long homology arm of 91 bp, and a short homology arm of 36 bp since asymmetrical ssODNs showed a higher efficiency of homology-directed repair using CRISPR/Cas9 [68].

**Table:** 

Sequence Name	Bases	Sequence
g1N141I guide RNA F	25	/5Phos/CACCGCATCATGATCAGCGTCATCG
g1N141I guide RNA R	25	/5Phos/AAACCGATGACGCTGATCATGATGC
Donor ssODN#A N141I	127	GAGAGAAGCGTGGCTGGAGGGCAGGGCCAGGGCCTCACCTTGTAGCAGCGGTACTTGTAGAGCACCACCAAGAAGATGGTCATAACCACGATGACGCTGATCATGATGAGGGTG**T**TCAGCACGGAGT

The donor sequence and pSpCas9-g1N141I-GFP vector were transduced in the AD1 and AD2 iPSC lines, plated at 50–70% confluency, using Amaxa Human Stem Cell Nucleofector kit (Lonza VPH-5002) and re-plated for recovery. GFP^+^ cells were sorted in a BD FACSAria IIu Cell Sorter and were seeded at 30–50 cells per well in 96-well format to detect and pick single clones. Positive clones were expanded, qDNA was extracted and analyzed for successful HDR was determined using a custom designed TaqMan genotyping assay with a probe specific for the SNP (dbSNP ID: rs63750215) located in Chr1:227,073,304 A > T. Selected clones were analyzed by Sanger sequencing to confirm correction of Chr1:227,073,304 location and discard possible insertions or deletions in the surrounding areas.

### Fluorescence-activated cell sorting (FACS)

Neural progenitors at day 12 of differentiation were dissociated with Accutase (Sigma-Aldrich) for 5 min at 37C and inactivated in Neurobasal media. Cells were spun at 1000 rpm for 4 min and the pellets were resuspended in FACS buffer (DPBS, 0.5% BSA Fraction V-Solution, 100 U/mL Penicillin-Streptomycin, 0.5% EDTA and 20 mM Glucose) with PE Mouse anti-human CD271 antibody (clone C40–1457, BD) at 1:100, also known as p75 or NGFR, and incubated for 20 min at room temperature (RT) in the dark. After the incubation time, cells were washed with FACS buffer and the pellet was resuspended in 2 mL of FACS buffer with 10 μM ROCK nhibitor (Y27632, Stemgent). p75 positive cells were purified in a BD FACSAria IIu Cell Sorter and data was analyzed using FlowJo software.

### Real-time quantitative polymerase chain reaction (RT-qPCR)

Human iPSC from PSEN2 mutants or control patients were grown in a monolayer and lysed directly in the cell culture wells with RLT buffer. Total RNA purification was performed with the RNeasy Micro kit (Qiagen), and was carried out according to the manufacturer’s instructions. cDNA was synthesized using SuperScript® III Reverse Transcriptase (RT) (Invitrogen, Carlsbad, CA). Semi-quantative real-time PCR was performed on StepOnePlus™ Real-Time PCR System (Applied Biosystems, Foster City, CA) using the primers listed in the table below. We normalized expression levels to GAPDH. The PCR cycling parameters were: 50 °C for 2 min, 95 °C for 10 min, followed by 40 cycles of 95 °C for 15 s and 60 °C for 1 min. Each biological variable was assessed in technical triplicates within each designated “Experiment”, and each designated “Experiment” was performed in at least three complete “start to finish” iterations. Expression levels were normalized to the control line, and results were expressed as AVG ± SEM.

**Table:** 

Gene	Forward Primer 5′ - 3’	Reverse Primer 5′ - 3’
*BDNF*	TAACGGCGGCAGACAAAAAGA	GAAGTATTGCTTCAGTTGGCCT
*BF1*	AGAAGAACGGCAAGTACGAGA	TGTTGAGGGACAGATTGTGGC
*Nkx2.1*	TAACGGCGGCAGACAAAAAGA	GAAGTATTGCTTCAGTTGGCCT
*NLRP2, ASB9*	From [77]	From [77]
*NLRP3*	ACGAATCTCCGACCACCACT	CCATGGCCACAACAACTGAC
*Tuj1*	GAAGTGTCCCAGGACATGATAA	CTCTTGAGTAGCTGGGATTGAG

### Aβ assays

Cells were conditioned for 3 days after day 8 of dual SMAD inhibition to measure secretion of Aβ by neural progenitors in vitro. Aβ levels were quantified using human/rat β amyloid 40 ELISA Kit and β amyloid 42 ELISA Kit high sensitive (Wako). Each biological variable was assessed using technical triplicates within each designated “Experiment”, and each designated “Experiment” was performed in at least three complete “start to finish” iterations.

### Immunostaining/ICC

Cells were fixed with PFA 4% directly on the wells of 12, 48 or 96 well plates for 20 min, washed 3 times with DPBS 1× (ThermoFisher). For the staining, cells were incubated in blocking solution (DPBS 1× with 0.1% Triton X-100 plus 5% Donkey serum) for two hours at room temperature. The corresponding primary antibodies were diluted at suitable concentration in blocking solution, and incubated overnight at 4C. The primary antibodies used are represented in (Additional file 1: Table S1). Cells were washed three times with DPBST (DPBS 1× + 0.1% Triton X-100) and suitable secondary antibody was added in blocking solution for 1 h at room temperature. Then cells were washed three times with DPBST and incubated with DRAQ5 or Hoescht 33342 (1 μg/mL, diluted in DPBS 1×) for 10 min at room temperature for nuclear counterstain. Cells were visualized using an inverted fluorescence microscope (Olympus IX71 microscope) or a confocal microscope (Zeiss LSM5 Pascal microscope) under 10×, 20× or 63× magnification. See Additional file 1: Table S1 for complete details of antibodies used in this study.

### Western blots

Human iPSC from PSEN2 mutants or control patients were grown in a monolayer and lysed directly in the cell culture wells with RIPA buffer (Thermo Scientific) with protease and phosphatase inhibitors. The protein concentration was measured using the BCA protein assay kit (Thermo Scientific). After protein estimation, 20 μg of cell lysate were separated by SDS-PAGE electrophoresis on a 4–12% Bis-Tris gel (Bolt® protein gels) and transferred onto nitrocellulose membranes by electrophoresis blotting. The membranes were blocked with blocking buffer 1X TBST (tris-buffered saline +0.1% Tween) plus 5% nonfat dry milk for 1 h in agitation at room temperature and washed three times with TBST. After washing, membranes were incubated at 4 °C overnight in agitation, with the primary antibodies against NLRP2 (1:1000), PSEN2 (1:200) or β-actin (1:1000). After rinsing, the membranes were incubated with horseradish peroxidase (HRP)-conjugated suitable secondary antibodies for 1 h at room temperature. Finally, protein bands were visualized with a chemiluminescent reagent according to the manufacturer’s instructions. β-actin was used as loading control.

### Electrophysiology

Whole cell patch-clamp recordings were obtained from single neurons between differentiation days 38 and 55. Cells were seeded at low density onto plastic coverslips which were placed in a perfusion based enclosed recording chamber. Neurons were localized using differential interference contrast optics under an Olympus BX61WI microscope fitted with a Hamamatsu Orca R^2^ CCD camera. Recordings were carried out at room temperature using MultiClamp 700B amplifier (Molecular Devices, Sunnyvale, CA, USA). Signals were sampled at 10 kHz and filtered at 6 kHz using a Digidata 1440A analog to digital converter (Molecular Devices). Amplifier control and data acquisition was done using pClamp 10.0 software (Molecular Devices).

During recordings neurons were perfused with oxygenated BrainPhys media (StemCell Technologies Inc). Medium resistance recording pipettes (4–6 MΩ) were filled with an intracellular solution consisting of (in mM) 130 K-gluconate, 10 KCl, 2 Mg-ATP, 0.2 Na-GTP, 0.6 CaCl_2_, 2 MgCl_2_, 0.6 EGTA, and 5 HEPES titrated to pH 7.1 and osmolarity of 310 mOsm. In some experiments, the intracellular solution also contained 4 mg/mL biocytin (Sigma-Aldrich) for post-hoc identification of individual neurons, which were visualized with streptavidin-conjugated Alexa 488 (Life Sciences) as described elsewhere [42]. After initial break-in, access resistance (Rs) was constantly monitored and recordings were discarded if Rs exceeded 20 MΩ or changed more than 30%. The voltage protocol for compound Na + and K+ currents characterization was as follows: cells were held at −80 mV potential followed by 500 ms steps from −100 mV to 30 mV with 10 mV increment at a frequency of 0.1 Hz. Following transition to current-clamp mode, resting membrane potential was recorded and cells were hyperpolarized by a negative DC current injection to −70 mV to ensure consistency of excitability measurements. Action potentials were evoked with square 1 s current steps from −10 pA to 40 pA with 1pA steps.

Electrophysiological recordings were analyzed using ClampFit software (Molecular Devices, Sunnyvale, CA, USA) and statistical significance of the results was measured using ANOVA test with Tukey’s post-hoc comparison of means. Salts and other reagents were purchased from Sigma-Aldrich (St. Louis, MO, USA).

### Statistical analysis

qPCR gene expression experiments and Aβ42/40 ELISAs were analyzed for statistical significance using Student *t-*test. LDH Release assays were analyzed by 2-Way ANOVA Bonferroni post hoc tests. ANOVA test with Tukey’s post hoc comparisons were used for analysis of electrophysiology results. The experiments needed to study each of the 94 neurons recorded for electrophysiology analyses required days to weeks. On each experimental day, representatives from each genotype were included, with at least three samples from each genotype studied on each day. *, *p* < .05; **, *p* < .01; ***, *p* < .001.

**Fig. 1:**
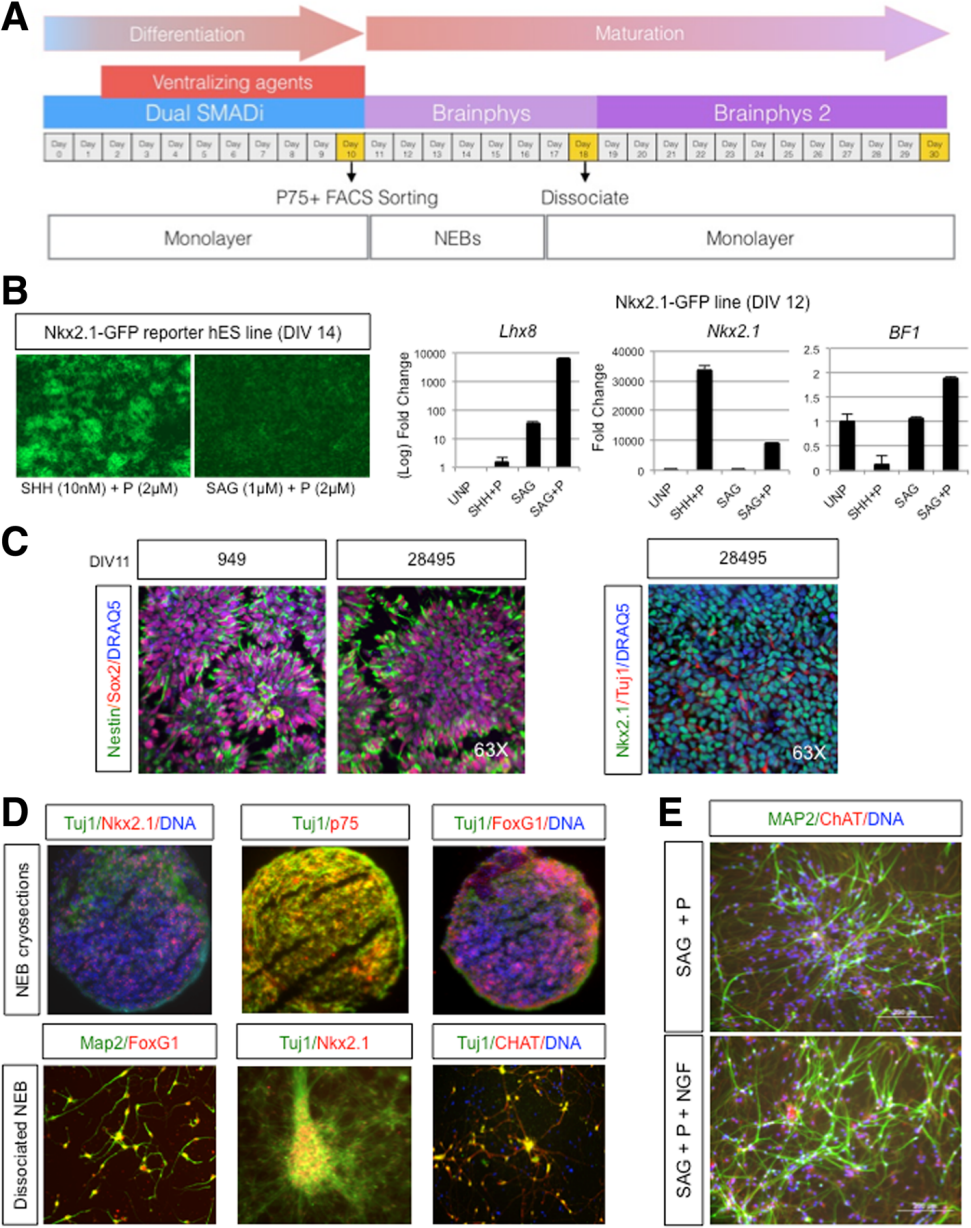
Overview schematic of basal cholinergic differentiation protocol. **a** Cells are plated and allowed to reach 100% confluency (Day 0), before the initiation of dual smad inhibition and the subsequent introduction of ventralizing agents (Day 2). At day 10 the monolayer is dissociated, sorted for p75+ cells, and kept as NEBs until day 19. Then the culture is dissociated again into a monolayer (See Methods for more details). **b** Left panel shows sustained EGFP expression driven by Nkx2.1 induction in NKx2.1-EGFP hESCs upon SHH plus purmorphamine or SAG plus purmorphamine treatment, maintained at Day 14, after removal of treatment at Day 8. Right panel shows *Nkx2.1*, *Lhx8* and *BF1* relative gene expression to GAPDH measured by qPCR, in NKx2.1-EGFP cell line in the presence of the indicated ventralizing agents, or unpatterned (UNP) at Day 12. *n* = 3, in technical triplicates. **c** Confocal microscope images of Nestin (green), Sox2(red) and DRAQ5 (blue) immunostaining in fControl and control lines at Day 11, showing typical neural rosettes (left panel), or Tuj1 (green), Nkx2.1 (red)right and DRAQ5(blue) in the right panel. Images representative of 3 independent experiments. **d** Fluorescence microscope images of immunostained NEB cryosections or dissociated NEBs into a monolayer with the BFCN markers Nkx2.1/Tuj1/p75/BF1/MAP2/ChAT. **e** Dissociated NEBs into a monolayer immunostained at Day 50 with MAP2(green), ChAT(red) and Hoescht (blue). Fluorescence microscope images the effect of NGF addition to SAG plus purmorphamine treatment alone. Images are representative of at least 3 independent experiments

## Results

### Optimization of protocol for BFCN differentiation

The scheme of BFCN differentiation is described in Fig. 1a. iPSCs from control subjects or AD patients were plated in feeder-free conditions and allowed to reach 100% confluency prior to differentiation using mTeSR1 basal media. Both branches of TGFbeta signaling were inhibited (dual SMAD inhibition) to induce neuroectodermal fate on “day 0” [12]. Differentiations (day 2–10) were performed using a modified mTeSR1 formulation, lacking factors that support pluripotency (bFGF, TGF-Beta, Li-Cl, GABA and pipecolic acid). To specify these cells to basal forebrain cholinergic neurons, ventralization for medial ganglionic eminences (MGE) induction is required [19, 85, 91]. As such cells were treated with the Sonic Hedgehog (Shh) analog (SAG) at 500 nM and Purmorphamine at 2 μM from days 2 to 8. SAG is a suitable substitute to activate Shh signaling, as demonstrated during differentiation of ChAT^+^ motor neurons and glutamatergic interneurons [91], with lower cost than recombinant Shh and some advantages in neuronal survival properties over Shh itself [7, 35]. We used the Nkx2.1-GFP embryonic stem cell (ESC) reporter line as a tool to adjust the combination, dosage and timing of ventralizing agents more beneficial for specification of BFCNs from induced Nkx2.1 basal forebrain precursors. However, given the potential of Nkx2.1 intermediate neural precursors to generate multiple neuronal subtypes, such as TH+ and GABA+ hypothalamic neurons, we analyzed the expression of the downstream cholinergic specification factor *Lhx8* over expression of the GABAergic interneuron specific transcription factor *Lhx6* [26] under different specification conditions (Fig. 1b). These data agree with those from [50] supporting the existence of a synergistic effect of SAG and purmorphamine on Nkx2.1 induction although an effect that is less than the effect of Shh plus purmorphamine (Fig. 1b). Nkx2.1-driven GFP levels were maintained after Day 14, even after withdrawal of SAG + purmorphamine at day 8 (Fig. 1b). We observed higher *Lhx8* induction upon SAG plus purmorphamine treatment than SAG alone, or even Shh plus purmorphamine (Fig. 1b). Interestingly, intermediate Nkx2.1 levels driven by SAG plus purmorphamine correlate with higher induction of *Lhx8* and BF1 gene expression (Fig. 1b). Our choice of starting SHH pathway-driven ventralization at day 2 was based on reports demonstrating other MGE-derived populations being generated by earlier (e.g., hypothalamic neurons) or later (e.g., GABAergic interneurons.) ventralization in the context of dual smad inhibition protocols.

Following the patterning stage, we gradually switched from Custom mTESR1 media to Brainphys media with B-27 supplement to support neuronal survival and growth [3]. At day 11, we observed neural rosettes positive for Nestin and Sox2 markers (Fig. 1c); also, we observed Tuj1+ neurites as early as day 11 (Fig. 1c). To obtain cholinergic populations of a higher purity, we developed a P75^+^ FACS strategy to isolate progenitors specific for cholinergic neurons due to the fact that BFCNs are the only CNS neuron type to express robust levels of P75 under non-pathogenic conditions in the adult brain). Support for this strategy includes a previously published protocol using FACS to isolate high expressing P75+ cells from the embryonic murine septum [65]. This population correlated with best expression of cholinergic-related markers.

At day11/12, we lifted the cells using chemical dissociation (Accutase) and purified day 11–12 p75+ (CD271) neural progenitors and generated 3D neuronal embryoid bodies (NEBs) by spinning down neural progenitors in V-bottom 96 well plates. On day 19 NEBs were dissociated and re-plated as a monolayer on plates coated with branched polyethylenimine (Aldrich catalog number 408727) and laminin. Monolayer cultures were maintained with the addition of growth factors BDNF, NGF and protease inhibitor DAPT until day 26, when cultures no longer had DAPT added. Immunostaining of both cryosections of NEB structures and fixed monolayers, resulting from chemical dissociation of NEBs from several control iPSC and H9 hESC lines, demonstrated expression of BFCN lineage markers Tuj1, MAP2, BF1, Nkx2.1 and p75, at final stages of the differentiation protocol (Fig. 1d). NGF addition to neuronal cultures showed an advantageous effect on maturation, neurite outgrowth and presence of ChAT (Fig. 1e).

### Generation and QC of *PSEN2*^*N141I*^ iPSC lines

**Fig. 2:**
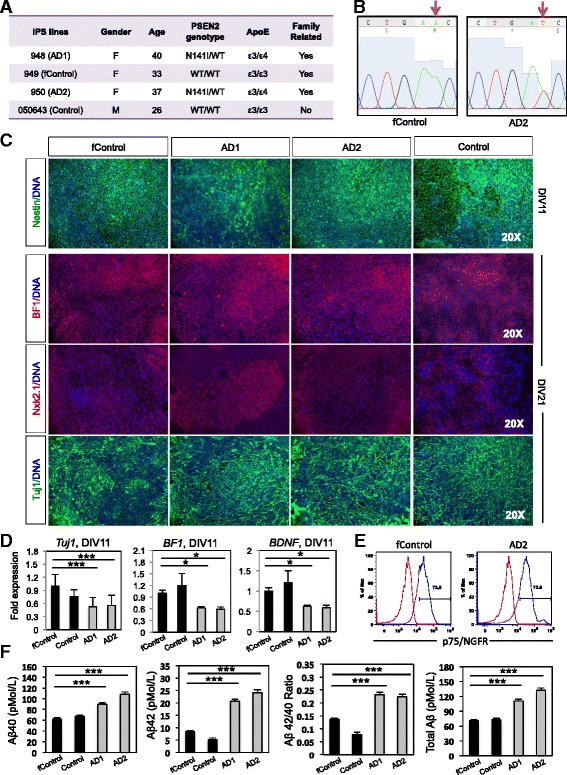
Basal cholinergic markers in *PSEN2* ^*N141I*^ neuroprecursors. **a** Table showing the cell lines used. Four iPS lines reprogrammed from fibroblasts were used; two controls (949 and 050643, labelled as fControl and Control, respectively) that do not carry the *PSEN2* ^*N141I*^ mutation nor the ε4 allele; and two AD patients (948 and 950, labelled as AD1 and AD2, respectively) who carry the mutation and the ε4 allele. Three of the four iPS lines were family related (fControl, AD1, and AD2). **b** Representative Sanger sequencing chromatograms showing a fragment of exon 5 of PSEN2. Red arrow marks site of the missense point mutation Chr1:227,073,304 A > T. **c** Immunocytochemistry and RT-PCR for early neuronal and basal forebrain markers. *n = 3,* 3 independent experiments with technical triplicates. **d** RTPCR fold changes for *TUJ1* and *BF1. n = 3,* 3 independent experiments with technical triplicates. **e** Representative histograms for P75 staining. *n* > 6. **f** Aβ40 and Aβ42 ELISA quantifications. *n = 3,* 3 independent experiments with technical triplicates. ***, *p* < .001. *, *p* < .05

*PSEN2* ^*N141I*^ mutant iPSC and control lines were generated from fresh skin biopsies. Established fibroblast lines were grown from skin punches donated by a kindred of 2 carriers for presenilin 2 Volga familial AD mutation (*PSEN2* ^*N141I*^) and one non-affected member. Additionally, we included a non-family related control. Fibroblasts were reprogrammed using modified RNA method to introduce the Yamanaka factors (Oct4, KLF4, SOX2 and c-Myc), and the iPSC lines obtained were subject to several quality control processes to ensure robust cell-renewal and pluripotency, including alkaline-phosphatase (AP) enzymatic activity, gene expression analysis and immunostaining for pluripotency markers, as well as karyotyping for detection of chromosome abnormalities, following the automated iPSC reprogramming and QC methods developed by [60]. A summary of the genotypes, sex and age of the subjects included in the study is shown in Fig. 2a. The two *PSEN2* ^*N141I*^ iPSC lines were also heterozygous for *APOE* ε4 (ε3/ε4), whereas the control iPSC lines were homozygous ε3/ε3. The characterization of the iPSC lines, expression of pluripotency markers and quality control results are shown in Additional file 2: Figure S1. Briefly, all iPSC clones selected demonstrated pluripotency by embryoid body formation and differentiation into the three germ layers (Additional file 2: Figure S1A). Finally, the lines were fingerprinted (Cell Line Genetics) to ensure they matched the parental fibroblast lines (data not shown). All the parental fibroblast lines and the iPSC lines were subject to Sanger sequencing to determine *PSEN2* and *APOE* genotypes. A 173 bp fragment from the exon 5 of *PSEN2*, surrounding the area where the *PSEN2* ^*N141I*^ point mutation is located (Chr1:227,073,304 A > T), was amplified by PCR and sequenced using the primers published in [53]; similarly a fragment of 244 bp from *APOE* locus that contains two SNPs which determine the three allelic variants was amplified by PCR from genomic DNA, and subsequently sequenced to distinguish between ε2/ε3/ε4 variants, using the primers from [36]. Sample chromatograms showing the presence of *PSEN2* ^*N141I*^ point mutation are shown in Fig. 2b, and all genotypes are summarized in Fig. 2a.

### Characterization of *PSEN2*^*N141I*^ neural progenitors

To study the effect of the *PSEN2* ^*N141I*^ mutation in early stages of the differentiation of cholinergic neurons, we analyzed the neural progenitors (NPCs) obtained at DIV 11–16 along the BFCN differentiation protocol. The analysis of this intermediate immature population allows us to detect possible early alterations in the generation of BFCNs that would otherwise not be detected in terminally differentiated cholinergic neurons. Such defects could potentially play roles in mature neurons and contribute to the pathophysiology of AD. We analyzed the expression of early neuronal markers in *PSEN2* ^*N141I*^ mutant and control NPCs by gene expression and immunofluorescence methods. Although, we found a lower RNA expression of Tuj1 (βIII-Tubulin), a general neuronal marker, in mutant NPCs at day 11 of differentiation, we did not detected quantifiable differences by immunocytochemistry circa days 16–21, (Fig. 2c and d). NPC monolayer cultures at day 11 were also immunostained for typical NPC markers: Sox2, and Pax6; with Pax6 levels dropping as expected along with Nkx2.1 induction (not shown). We observed comparable expression of Sox2 and Nestin in *PSEN2* ^*N141I*^ cultures at day 11 (Fig. 2c, top panel). At day 21, mutant NPCs expressed comparable levels of Nkx2.1 (MGE marker), but reduced levels of BF1 (forebrain marker) by qPCR; however, BF1 protein expression did not seem affected by immunostaining at this differentiation stage (Fig. 2c bottom panel, and d). We did not observe differences in the surface expression of NGFR (p75/CD271) in DIV11–12 *PSEN2* ^*N141I*^ cells, in terms of percentage of positive cells or fluorescence mean peak value (Fig. 2e).

As previously published by [59, 73], the expression of mutant *PSEN2* ^*N141I*^ causes an increase in the Aβ42/40 ratio in the brains of transgenic mice; additionally, this enhanced Aβ42 production was observed in neural cell lines upon induced overexpression of mutant *PSEN2* ^*N141I*^ protein [83] and in iPSC derived from *PSEN2* ^*N141I*^ mutant patients [93]. Consistently, we observed a 2-fold increase in the Aβ42/40 ratio, a 50% increase in the amount of secreted Aβ40 and 2.5-fold increase in Aβ42 species in the conditioned media from *PSEN2* ^*N141I*^ neural progenitors at DIV 11 (***p < 0.001) (Fig. 2e). The levels of secreted Aβ40 and 42 observed in our study and the levels found in [93], using a different neuronal differentiation method applied to FAD1/PS2 iPSC lines derived from fibroblasts from the Coriell repository are very similar in both absolute number and in fold-increase.

### Characterization of mature BFCNs from *PSEN2*^*N141I*^ iPSC lines and controls

**Fig. 3:**
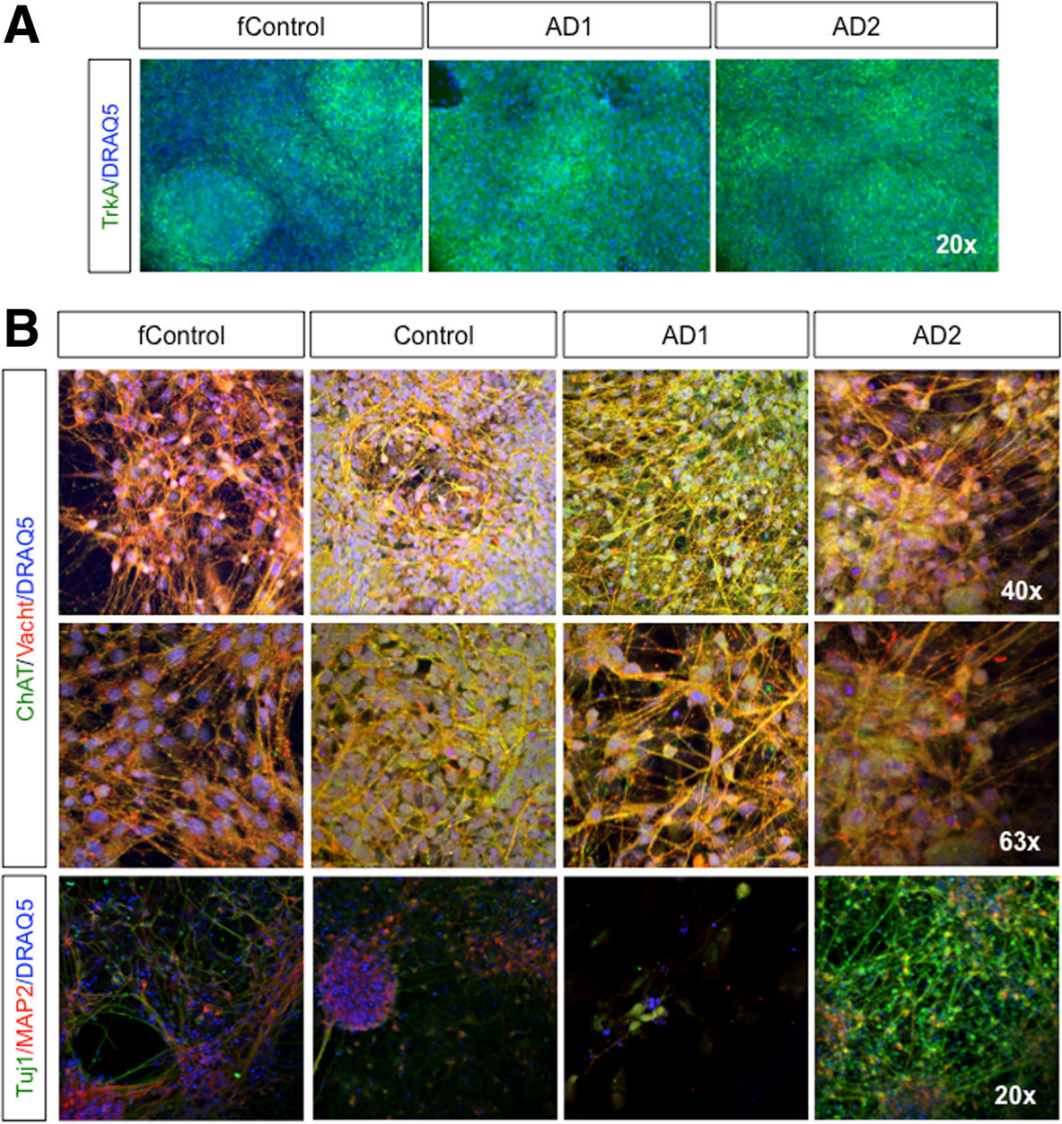
Neuronal and basal cholinergic markers by immunocytochemistry. **a** Immunostaining for TrkA on DIV 21. **b** Immunostainings for ChAT and vAChT at different magnifications at DIV65; and Tuj1 and MAP2. Images are representative of at least 3 independent experiments

With the aim of determining the impact of *PSEN2* ^*N141I*^ mutation on the differentiation, gene expression, function, and communication of BFCNs, we characterized cells at later time points for appropriate expression markers; our goal was to explore whether *PSEN2* ^*N141I*^ iPSC were able to complete BFCN maturation process and if so, if any abnormalities along later stages of BFCN differentiation may account for the pathophysiology of EOFAD (Fig. 3). In addition to p75, which preferentially binds pro-NGF, we analyzed the expression of TrkA, the primary mature NGF receptor, was also expressed in *PSEN2* ^*N141I*^ BFCNs and control (Fig. 3a). This suggested that *PSEN2* ^*N141I*^ BFCNs are susceptible to receiving and benefiting from NGF pro-survival and differentiation signals as expected and further confirms their proper identity. We observed comparable expression of additional cholinergic neuron specific markers choline acetyltransferase (ChAT) and vesicular acetylcholine transporter (vAChT) in *PSEN2* ^*N141I*^ BFCNs and controls (Fig. 3b). Other general neuronal markers such as Tuj1, and the mature marker microtubule-associated protein 2 (MAP2) showed no apparent differences by immunofluorescence (Fig. 3b).

### CRISPR/Cas9-mediated correction of *PSEN2*^*N141I*^ mutation and effect on Aβ 42/40 ratio

**Fig. 4:**
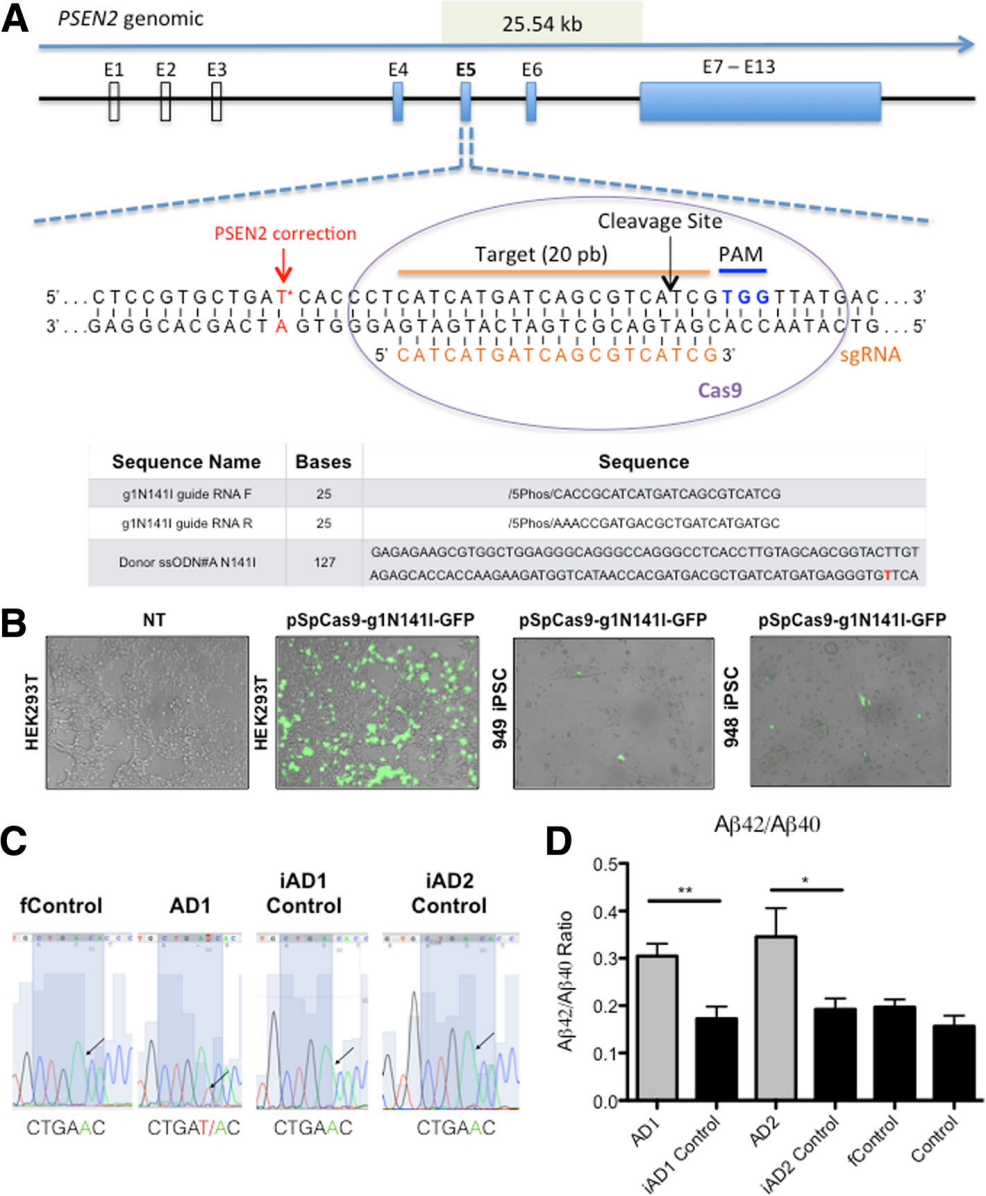
CRISPR/Cas9-mediated correction of *PSEN2* ^*N141I*^ iPS lines. **a** Schematic showing guide RNAs used in the targeting of CRISPR/Cas9, as well as donor ssODNs utilized to introduce wild-type genotype. **b** Left 2 panels show GFP positive HEK293T cells indicating Cas9 system with guide RNA expression, NT refers to non-transfected; right 2 panels show sample of GFP positive iPSCs after lipofection with pCas9-gN141I-GFP vector. **c** Sanger sequencing results from iPSC lines, showing corrections in the N141I mutation. **d** Aβ 42/40 ratio detected by ELISA in 72 h conditioned media from mutant, control or Cispr-Cas9 corrected BFCNs (DIV 34). *n = 4*, 4 independent experiments with technical triplicates. *, *p* < .05; **, *p* < .01 Student *T*-test

To determine if the molecular alterations in the processing and cleavage of APP and/or the exacerbated activation of NLRP2 inflammasome, as previously observed in PSEN1 mutants [77], can be attributed to *PSEN2* ^*N141I*^ mutation only, we modified the *PSEN2* locus in our iPSC lines employing CRISPR/Cas9 technology. We did this by correcting the *PSEN2* ^*N141I*^ point mutation in the two *PSEN2* mutant iPSC lines (AD1, AD2). For this purpose, a specific guide RNA (g1N141I) was designed using an online tool (http://tools.genome-engineering.org) to direct Cas9 to the region of *PSEN2* exon 5 surrounding *PSEN2* ^*N141I*^ mutation (23 bp upstream of Chr1:227,073,304 A > T). g1N141I was cloned into pSpCas9(BB)-2A–GFP (PX458) vector. Expression was assessed by GFP fluorescence upon transfection of pSpCas9-g1N141I-GFP in HEK293T (Fig. 4a).

In order to correct the mutation, we designed an asymmetric ssODN HDR (homology directed repair) template, ssODN#A-N141I, with a long homology arm of 91 bp, and a short homology arm of 36 bp since asymmetrical donor sequences with a shorter arm oriented to the area closer to the PAM side demonstrated a superior efficiency of homology-directed repair using CRISPR/Cas9 system [13]. We then proceeded to transduce pSpCas9-g1N141I-GFP and ssODN#A-N141I into the iPSC lines using Amaxa nucleofection (Fig. 4a). Forty-eight hours post-nucleofection cells were dissociated and the GFP^+^ population was purified by FACS and replated at low density feeder free for isolation of single gene-corrected clones (Fig. 4b). Subsequently, clones were grown and gDNA extracted post expansion. The screening of positive clones that demonstrated successful HDR was determined by qPCR using a custom designed TaqMan genotyping assay with a probe specific for the SNP (dbSNP ID: rs63750215) located in Chr1:227,073,304 A > T. We were able to distinguish by this method between homozygous *PSEN2* ^*N141I*^, heterozygous *PSEN2* ^*N141I*^ and *PSEN2* ^*WT*^ single clones derived from the original iPSC lines, and pre-selected clones were subjected to Sanger sequencing to confirm Chr1:227,073,304 location and detect possible insertions, deletions or mismatches introduced by CRISPR/Cas9 modification in the surrounding area and corroborate successful HDR (Fig. 4c).

Successfully corrected clones were expanded and subjected to the BFCN differentiation protocol in parallel to the other 4 lines used in the study. We collected media from BFCNs (DIV 34) and re-tested for amyloid beta production. In support of our previous finding in NPCs at DIV11–12 (Fig. 2f), we observed that mature BFCNs also display significant increases in Aβ42/40 ratio (Fig. 4d) and overall Aβ production (Additional file 3: Figure S2). Importantly, these results also showed a normalization of Aβ42/40 ratio to control levels in corrected lines (iAD1 Control and iAD2 Control, are corrected clones of AD1 and AD2, respectively) (Fig. 4d). These results also strengthen previous findings linking the *PSEN2* ^*N141I*^ mutation to abnormal APP processing and reinforcing that presenilins contains the catalytic site of γ-secretase [90].

### Assessment of sensitivity to Aβ42 oligomer toxicity in iPSC-derived *PSEN2*^*N141I*^ neurons

**Fig. 5:**
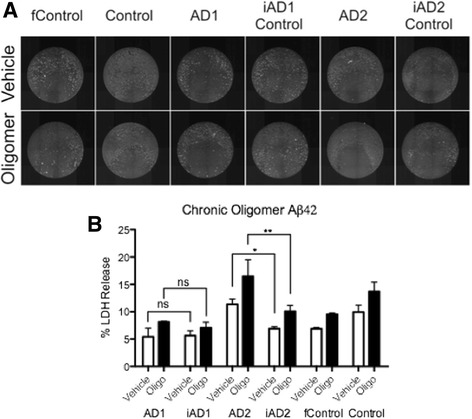
BFCNs carrying various *PSEN* mutations are not consistently more susceptible to Aβ42 oligomer toxicity. **a** Sample images of BFCNs from the indicated genotypes treated with propidium iodide to visualize cell death in response to 72-h exposure to Aβ42 oligomers (5 μM). **b** % LDH Release recorded from media collected after 72-h exposure. *n = 3,* 3 independent experiments with technical triplicates. *, *p* < .05; **, *p* < .01 as detected by 2-Way ANOVA Bonferroni post hoc tests

Previous reports have shown that iPSC lines carrying FAD mutations may display an enhanced susceptibility to noxious stimuli, such as high concentrations of Aβ42 oligomers [2]. We therefore tested whether our BFCNs from *PSEN2* ^*N141I*^ mutants would display enhanced toxicity to Aβ42 oligomers in the media (Fig. 5). We assessed neurotoxicity by measuring the percentage of lactate dehydrogenase (LDH) released by dead cells, thus providing an indirect measurement for toxicity. Using this methodology by 2-way ANOVA we detected a significant effect in toxicity driven by 5 μM Aβ42 oligomer addition to the culture media, after 72-h exposure (***, *p* < 0.01). Post hoc Bonferroni analysis revealed significant differences between the AD2 line and its corrected isogenic control (iAD2 Control). However, this apparent enhanced sensitivity to Aβ42 oligomer toxicity was not observed in the AD1 line and its corresponding control. These results indicate that differences in susceptibility to Aβ42 are not exclusively linked to mutant *PSEN2* genotype, and that likely additional genetic factors different between AD1 and AD2 subjects affect susceptibility to this stress, further emphasizing the importance of multiple isogenic models.

### Assessment of NLRP2 mRNA in iPSC-derived *PSEN2*^*N141I*^ neurons

**Fig. 6:**
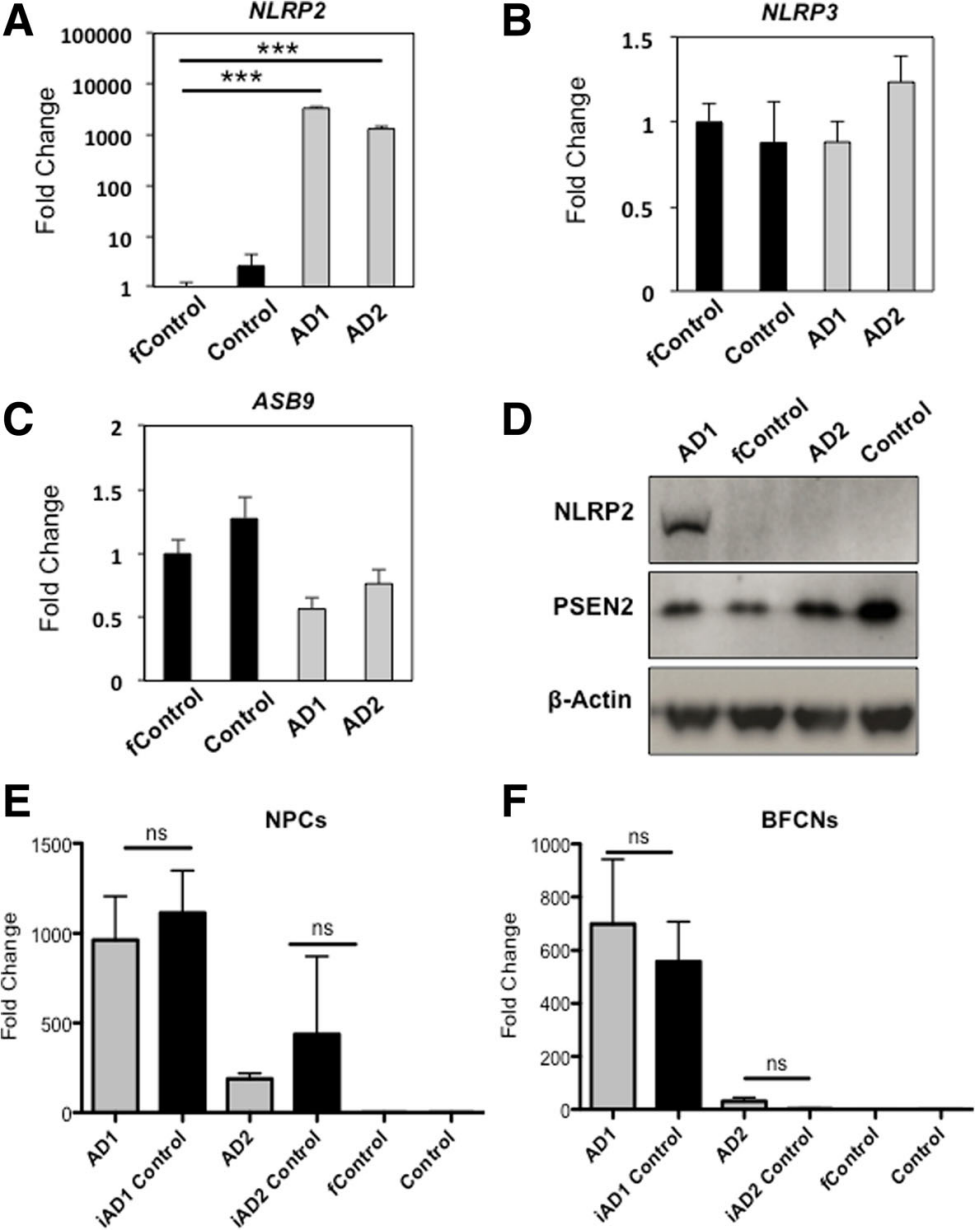
NLRP2 inflammasome mRNA levels are over-expressed in some *PSEN2* ^*N141I*^ cells, but it is not driven by mutation. RT-PCR expression of (**a**) *NLRP2*, (**b**) *NLRP3*, and (**c**) *ASB9* in cholinergic neuroprecursors. **d** Western blot showing NLRP2, PSEN2 and β-Actin. RT-PCR expression of *NLRP2* in Neuroprecursors (**e**) and BFCNs (**f**). *n = 3*, 3 independent experiments with technical triplicates, for all panels. ***, *p* < .001

We previously reported that *NLRP2* mRNA was elevated in *PSEN1* mutant iPSC and NPCs, [77] which was also the case for *PSEN1* mutant cortical neurons (unpublished observation). Therefore, we wanted to analyze the status of the components of the inflammasome in the context of *PSEN2* ^*N141I*^ mutation. When we assayed by qPCR the mRNA levels of *NLRP2* in NPCs at DIV12, we observed an increase over 100-fold in AD1 and AD2 lines, as compared to control lines (Fig. 6a). This correlated with a notable increase in NLRP2 protein, as observed by SDS-PAGE in whole cell lysates from day 11 *PSEN2* mutants (Fig. 6d). Noticeably, however we did not detect bands for *NLRP2* by immunoblot in the AD2 line lysates. Further, we were unable to corroborate some other transcriptional events previously seen in *PSEN1* mutant iPS neural precursors, such as the elevated *ASB9* that encodes an E3 ligase that directs mitochondrial creatine kinase for degradation. Instead, we observed a trend toward decreased levels in *PSEN2* mutation carriers by 20–30%.

### Assessment of excitability of iPSC-derived *PSEN2*^*N141I*^*BFCNs*

Using BFCN differentiation protocol, we were able to generate electrophysiologically active cholinergic neurons in a dish from two *PSEN2* ^*N141I*^ mutant AD patients, wild-type and familial controls starting from differentiation day 35. We were initially unable to obtain mature action potential waveforms from BFCNs grown in Neurobasal media at this stage, but switching to BrainPhys media significantly improved electrophysiological properties of cultured neurons [3]. These findings are in line with electrophysiological characterization of other iPSC generated neurons used to compare both media [3]. We have also replicated the benefits of our protocol containing BrainPhys media in two additional cell lines (including the H9 embryonic stem cell line) with comparable endpoint expression of ChAT and VAChT as well as electrophysiological responses (data not shown).

**Fig. 7:**
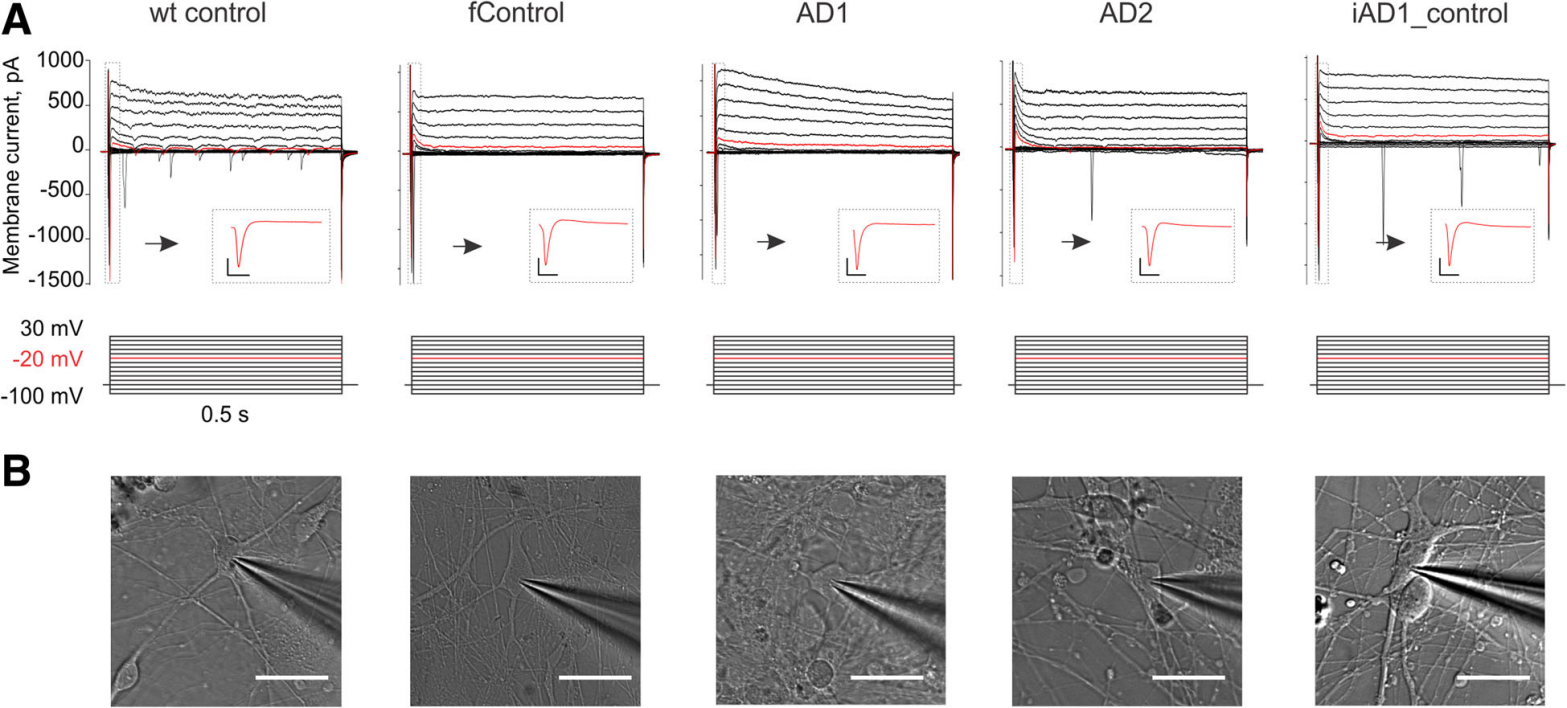
Electrophysiological and morphological features of BFCN. **a** Top row – compound sodium and potassium currents produced by a voltage protocol shown in bottom row. Current trace produced by a voltage step to −20 mV shown in red. Inset shows first 25 ms of a current produced by a voltage step to −20 mV (scale bars 200 pA, 5 ms). **b** Differential interference contrast image of a patched BFCN recorded in **a**. Ninety-four neurons (22 wild-type control, 21 familial control, 18 AD1, 28 AD2 and 5 iAD1_control). Scale bar is 30 μm

**Fig. 8:**
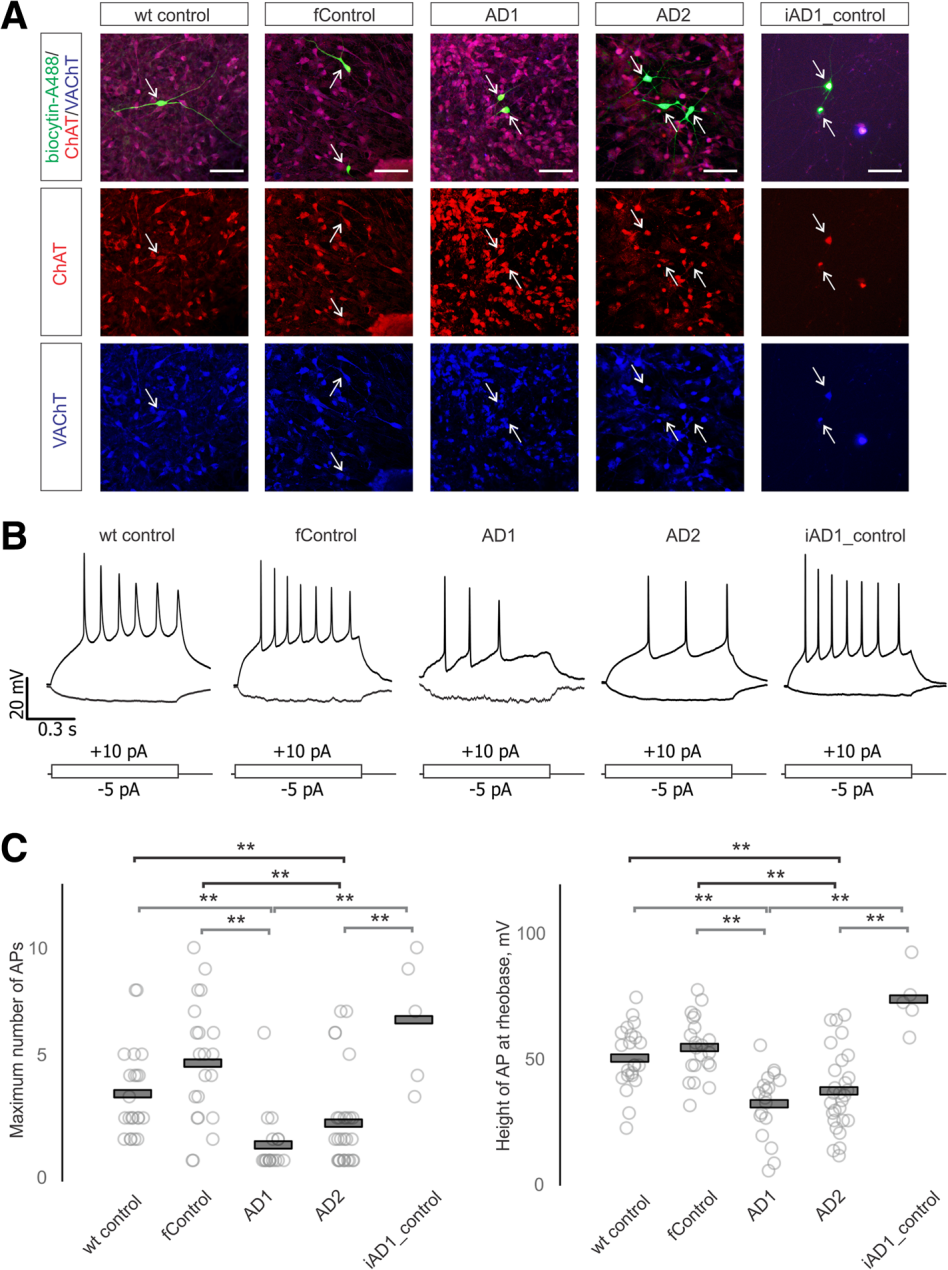
Electrophysiological deficits in BFCNs from AD lines. **a** Co-localization of biocytin-labelled neurons (green) with cholinergic markers ChAT (red) and VAChT (blue). Arrows indicate positions of recorded neurons somas, scale bar is 50 μm. **b** Representative firing patterns of BFCNs produced by a 1 sec negative and positive square current injection are depicted. A grand total of 94 individual neurons were studied electrophysiologically: 22 wild-type control neurons, 21 familial control neurons, 18 AD1 neurons, 28 AD2 neurons, and 5 iAD1_ (CRISPR-corrected) neurons. The experiments on the 94 neurons required days to weeks. On each experimental day, representatives from each genotype were included, with at least three samples from each genotype studied each day. **c** Summary data on maximum number of action potentials that neurons are capable of sustaining (left) and height of a single action potential at rheobase (right) across all conditions. Individual data points are shown as circles, group means are shown as bars. **, *p* < 0.01 Tukey HSD test

In order to investigate the electrophysiological properties of BFCN, we recorded from a total of 94 neurons (22 wild-type control, 21 familial control, 18 AD1, 28 AD2 and 5 iAD1_control) using whole cell patch-clamp method. In all experimental groups, recorded neurons displayed voltage-activated currents through sodium and potassium ion channels, ability to generate action potentials and displayed classical neuronal morphologies (Fig. 7). In subset of experiments, recorded neurons were labeled with biocytin through a patch pipette, which allowed for post hoc cell identification and ICH characterization. We found that all biocytin-labelled cells were also immuno-positive for ChAT and VAChT (*n* = 12, Fig. 8a).

**Fig. 9:**
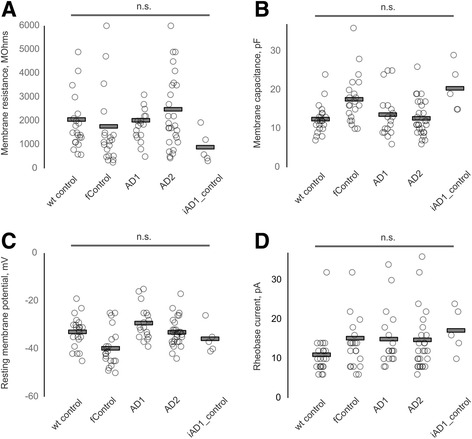
Intrinsic electrophysiological properties of BFCNs. Summary data on all recorded BFCNs from five groups. Ninety-four neurons (22 wild-type control, 21 familial control, 18 AD1, 28 AD2 and 5 iAD1_control). Histograms show individual values from each neuron (circle) and group means (bars) for membrane resistance (**a**), capacitance (**b**), resting potential (**c**) and rheobase current (**d**). Statistical significance was tested with ANOVA and Tukey’s post hoc comparisons

We did not observe significant differences between the groups in terms of neuronal membrane resistance and capacitance, membrane resting potential and the minimum current required for generation of a single action potential (Fig. 9). However, we observed significant mutation-related, editing-reversible differences in excitability of BFCNs. Neurons derived from AD1 and AD2 lines (as compared to WT and familial controls) were able to generate fewer maximum number of spikes in response to a square depolarizing current injection (ANOVA test with Tukey’s post hoc comparisons, Fig. 8b, c). Height of the first action potential at rheobase current injection was also significantly decreased in AD1 and AD2 BFCNs (Fig. 1c). Importantly, CRISPR/Cas9 correction of the *PSEN2* point mutation in the AD1 mutant iPSC line abolished the observed electrophysiological deficit, restoring both the maximal number of spikes and spike height to the levels recorded in wild-type and familial controls (ANOVA test with Tukey’s post hoc comparisons, Fig. 8)*.*

## Discussion

There are 5 million people currently affected by Alzheimer’s disease in the US and, according to the Alzheimer’s Association, this number will increase to 16 million by the year 2050. Unfortunately, we only have direct evidence for genetic causation that accounts for 3–5% of these patients. This percentage encompasses the EOFAD variants, caused by inherited fully penetrant autosomal dominant mutations in the amyloid protein precursor (*APP*), or *PSEN1*, *PSEN2* that constitute the γ-secretase apparatus [87], and changes in their function increases the production of Aβ42 oligomers and/or deposition of amyloid plaques.

After decades studying murine models of AD that do not fully recapitulate the pathophysiology of this disease in the human brain [5, 57, 58], a complementary new concept of AD modeling in vitro has emerged upon the breakthrough by [81] allowing adult human tissue reprogramming into iPSC using defined factors, and their subsequent in vitro differentiation into specific brain cell types.

BFCNs are one of the most vulnerable neuronal populations whose deterioration explains, in part, the cognitive decline in AD patients. Apart from the evidence for BFCN failure and atrophy, other studies have revealed that human embryonic stem cell-derived BFCNs transplanted into AD mouse models can be associated with improvement in the learning behavior of the implanted mouse [94]. These findings highlight the relevance of iPSC- and ESC-derived BFCNs as not only early clinical indicators but also as a potential strategy for subtype-specific cell-based therapy for AD [39]. In order to move this cell-based therapeutic strategy forward, there has been an urgent need for a refined differentiation protocol to generate human ESC- and/or iPSC-derived BFCNs.

Our first goal was to develop an improved protocol for the generation of BFCNs and intermediate neural progenitors (NPCs), followed by the use of these methods when differentiating cell lines from both control subjects and those harboring the *PSEN2* ^*N141I*^ mutation. Using fibroblasts isolated from 3 sisters, 2 carrying the PSEN2 mutation and displaying cognitive decline, with the third wild-type for the mutation, iPSCs were developed [60]. In order to approach the dissection of the fidelity of linkage of various phenotypes to the pathogenic mutation, we began by optimizing published BFCNs protocols [4, 17, 46, 50, 89] including the purification of an intermediate CD271^+^ (p75) forebrain progenitor population by Fluorescence Activated Cell Sorting (FACS) to generate 3D ventralized neural embryoid bodies (NEBs), which can be later dissociated to look at neuronal populations in a monolayer.

After induction of BFCN differentiation in these cell lines, we have analyzed: (1) capacity to generate Tuj1^+^/BF1^+^/ChAT^+^ neurons in vitro; (2) expression of genes/proteins of interest related to neuronal differentiation or inflammation; (3) generation of soluble and oligomeric A*β*40 and 42; (4) electrophysiological (ePhys) properties; and (5) selective vulnerability of BFCNs to one or more innate or microenvironmental factors within or in close approximation to those cells.

Several studies in AD mouse models highlight electrophysiological defects associated to late stages of AD pathology. Synaptic function in the hippocampus was reduced in APP23 mouse models [70]. Similarly, cholinergic neurons from the prefrontal cortex of TgCRND8 mice are unable to sustain cholinergic excitation as compared to control mice [64]. Here we report deficient electrophysiological properties in *PSEN2* ^N141I^ iPSC-derived BFCNs in vitro. Notably, correction of this point mutation re-established neuronal excitability to the level of the control iPSC-derived neurons.

We have optimized an in vitro BFCN differentiation protocol from human iPSC, focusing on generating a homogeneous population of electrophysiologically active ChAT+/VAChT+ neurons in a reproducible and fast way. The innovations introduced to the protocol granted a homogeneous expression of Nkx2.1, a transcriptional marker for MGE subregions, as soon as day 8 and very robust by day 11, compared to day 20 suggested in previously published protocols [38]; in defined serum-free media conditions and without forcing overexpression of factors implicated in cholinergic fate. We were able to record mature action potentials in neurons from day 38 in culture, accompanying co-expression of cholinergic specific markers, which is an earlier time point as compared to other existing protocols using ES or iPSC [4, 17, 46, 50, 89]. Therefore, our protocol has potential application to high-throughput drug screening in homogeneous cholinergic cultures. In addition, the 3D structure of NEBs themselves if left undisssociated organoid form would also allow mechanistic analysis in a more physiological setting.

After applying this optimized protocol to *PSEN2* ^*N141I*^ mutant iPSC lines, we found an increase in Aβ42/40 ratio in the conditioned media. We did not observe any evident defects in the neuronal differentiation process and expression of BFCN markers. Interestingly, we observed a decrease on BDNF gene expression in *PSEN2* ^*N141I*^ NPCs, similar to results described in a report [18] wherein BDNF changes were observed in homozygous and heterozygous *APP* ^*swe/*^ *PSEN1* ^*M146V*^ mice. The two mutant lines are also carriers of one *APOE ε4* allele. The presence of this allelic variant, the most common and well characterized risk factor polymorphism for LOAD [16], may modulate the age of onset and severity of the phenotype [49]. Therefore, these iPSC lines combining both the EOFAD *PSEN2* Volga mutation (or CRISPR/Cas9 corrected) and *APOE* ε4 allele constitute a tremendously useful tool to study the pathophysiology of early onset AD in vitro, especially when apoE-secreting iPSC-derived astrocytes are also present.

Searching for adjacent mechanisms or events that may be a cause or a consequence of elevated β-amyloid production, researchers have found overactivated inflammation and electrophysiological defects associated with AD mutations. The concept of these defects being independent from β-amyloid deposition and their demonstration using CRISPR/Cas9 technology to correct EOFAD mutations would open the debate to the need of combined AD treatments not only targeting β-amyloid plaques (Gandy et al.*,* in press), but also to overcome parallel inflammatory processes or excitotoxicity/defective neuronal firing.

NLRPs are components of the inflammasome, which induces the secretion of mature pro-inflammatory cytokine IL-1β in response to pathogens and toxic stimuli [11, 41]. NLRP2 appears dysregulated in astrocytes [45, 51], and NLRP3 in microglia [34] in the context of Alzheimer’s disease as well as in other neurological diseases like Parkinson’s disease [14, 32]; additionally, NLRP2/3 are altered in pathologies that show comorbidity with AD: obesity, type-2 diabetes. We previously reported an unexpected association of elevated expression of the inflammasome gene *NLRP2* in iPSC-derived neurons from banked fibroblasts from subjects harboring *PSEN1* ^*A246E*^ and *PSEN1* ^*M146L*^ mutations [77]. This association reminded us of the association of the inflammatory skin disease acne inversa (AI) with mutations in presenilin 1, nicastrin, APH-1 and PEN-2, raising in our minds the question of whether some γ-secretase component mutations might be associated not only with proamyloidogenic actions but also with proinflammatory mechanisms.

Despite our observations *PSEN2* ^*N141I*^ mutant cells had elevated NLRP2 compared to controls, we were not able to attribute this upregulation to the familial *PSEN2* mutation, as gene correction did not significantly reduce NLRP2 levels. Our results suggest that, although inflammasome dysregulation may occur in the brains of EOFAD patients, there may be factors triggering this event apart from any effect of *PSENs* on inflammasome biology that are reflected in reprogrammed PSEN2 mutant cell lines. Some potential explanations for this PSEN2-independent NLRP2 upregulation include effects of the apoE4 allele present in both PSEN2 subjects (not preset in controls) or epigenetic effects on fibroblasts collected from the EOFAD subjects that are maintained through the reprogramming process.

Electrophysiological defects in neurons have been associated with *PSEN1* and *PSEN2* mutations. Some of these defects are attributed to altered function of voltage-gated K+ channels, potentially through the cleavage of channel components mediated by the PS/γ-secretase apparatus [44, 72]. Presenilin mutations also disrupt calcium signaling by increasing the levels of calcium stored in the endoplasmic reticulum that result in increased stimulus-induced released into the cytosol, rather than altered influx of calcium. One of the mechanisms behind neuronal calcium dysregulation was described in cortical neurons from *PSEN1* ^*M146V*^ mice, mediated by inositol triphosphate (IP3) [79]; and, more directly, the formation of dual function protein-ion channels by unprocessed PSEN1 and PSEN2 themselves, modulating the exit of calcium from the endoplasmic reticulum [29, 55, 80, 84]. Given the important role of presenilins on potassium and calcium flux and neuronal excitability, mutations in *PSEN1* and *PSEN2* may lead to reduced neuronal excitability and neurotoxicity. Mice carrying mutant forms of APP exhibited aberrant action potentials associated to a decrease in sodium currents with no alteration in potassium currents, only after plaque burden was considerable [9]. There is evidence that APP overexpression causes hyperexcitability in mouse cortical neurons [75, 86, 92].

Mucke and Selkoe [52] have highlighted a toxic effect of Aβ resulting in synaptic and network dysfunction. In fibroblasts and neural cell lines, Aβ-mediated accumulation of mitochondrial Ca^2+^ was elevated when mutant forms of PS1 were expressed [31]. Neuronal firing patterns in mouse hippocampal neurons were altered by exposure to Aβ [67, 69]. Aβ exposure was also associated with altered K^+ ^ channel conductance in pyramidal neurons [54]. *PSEN1* mutations have been observed to associate with altered Ca^2+^ mitochondrial channels in the cerebellum, apparently causing reduced spike activity in Purkinje cells in the absence of amyloid plaque deposition [74]. Aβ42 may accentuate the defects present in Ca^2+^ homeostasis by modulation of additional voltage-dependent ion channels [8, 25, 76, 88].

Apart from mouse data and immortalized neuronal cell lines, electrophysiological defects in iPSC-derived neurons upon exposure to Aβ have been shown: hiPSC-derived cortical pyramidal neurons and GABAergic interneurons have deficient action potentials upon exposure to Aβ [56], and neurons differentiated from hiPSC harboring *PS1* ^*A426E*^ mutation also showed deficient firing patterns [47]. However, there are no previously published data on characterization of electrophysiological properties of *PSEN2* mutant iPSC-derived BFCNs.

Hyper- or hypoexcitatory effects and differences in firing frequency vary with the gene mutation and are highly dependent on the neuronal subtype [37, 48]. All these events may contribute to the progressive neurodegeneration present in the pathogenesis of AD, and we specifically document events that may account for the neuronal defects associated to early stages of EOFAD human pathogenesis. Here we report defective electrophysiological properties in iPSC-derived BFCNs that are specifically associated with the *PSEN2* ^N141I^familial mutation. Interestingly, although some of the previous studies attribute this impairment in neuronal activity to the build-up of plaques in the brain of AD mice, we found a substantial impairment in the induced action potentials in the absence of amyloid plaques, solely in the presence of an discrete excess of Aβ42 oligomers in the culture media, consistent with other reports [18]. Correction of this point mutation re-established the firing patterns to those of the wildtype iPSC-derived neurons.

Modulators of potassium channels in neurons have proven efficacy in memory improvement in AD mouse models [44]. Modulation of Ca^2+^ channels and excitotoxicity may open a new wave of AD drugs. Understanding the mechanism through which *PSEN2* mutations affect the electrophysiological activity in different subsets of neuronal populations and unraveling the connection between *PSEN2*, other genetic modulatory factors and inflammation will potentially lead to, not only alternative symptomatic treatments, but also to novel drugs decreasing the Ca^2+^-mediated vulnerability to ROS and potentially stopping the neuronal loss and progression of the disease, if administered at early stages.

It is clear that mutant presenilins alter neuronal excitability even before the formation of Aβ plaques [18, 74]. One plausible hypothesis is that APP and presenilins may exert effects that modulate neuronal excitability through currently unrecognized mechanisms acting separate from their roles in the biogenesis of Aβ. Accumulation of Aβ could synergize with the altered electrophysiological mechanisms in a pathway leading to AD. With the wealth of data supporting neuronal excitotoxicity as a key mechanism implicated in AD, further studies focusing on clarifying the possible role(s) of *PSENs* and/or Aβ in physiological or pathological events are warranted.

## Conclusions

We have optimized an in vitro protocol to generate human BFCNs from iPSCs from presenilin 2 (*PSEN2)* mutation carriers and controls. As expected, *PSEN2* ^*N141I*^ was associated with an increase in the Aβ42/40 in iPSC-derived BFCNs, and this was reversed by CRISPR/Cas9-mediated gene editing. Unexpectedly, iPSC-derived BFCNs or cortical neurons from *PSEN2* ^*N141I*^ carriers showed diminished basal excitability as quantified by a reduction of both spike frequency and spike amplitude. This electrophysiological phenotype was also abolished following CRISPR/Cas9 correction of the *PSEN2* ^*N141I*^ mutation. The gene editing data confirm that there was a robust consistency of mutation-related changes that characterized all the expected findings and genotypes from all cells.

## Additional files

Additional file 1: Table S1. Antibodies, Species, Titers, and Vendors Used in this Study. (DOC 31 kb)

Additional file 2: Figure S1. Quality control of iPSC lines. (A) Immunofluorescence shows expression of pluripotency markers SSEA4, Nanog, Tra160 and in 7889(S)B iPSC line. (B) Three germ layers from teratomas generated by 7889(S)B iPSC line. (TIFF 2702 kb)

Additional file 3: Figure S2 Amyloid β levels in mature BFCNs. (A) Levels of Aβ40 on BFCNs (DIV 34). *, *P* < .01 vs. other lines in study according to One-Way ANOVA Bonferroni Post-hoc test. (B) Levels of Aβ42 on BFCNs (DIV 34). *n = 3*, 3 independent experiments with technical triplicates. *, P < .01 based on Student’s T-test. (TIFF 1753 kb)

## Abbreviations

AD: Alzheimer’s disease
ApoE: Apolipoprotein E
APP: Amyloid protein precursor
AVG: Average
Aβ: Amyloid beta
BDNF: Brain derived neurotrophic factor
BF1: Brain factor 1
BFCNs: Basal forebrain cholinergic neurons
ChAT: Acetylcholine transferase
DAPT: (N-[N-(3,5-difluorophenacetyl)- L-alanyl]-S-phenylglycine t-butyl ester)
DIV: Days in vitro
DNA: Deoxyribonucleic acid
DPBS: Dulbecco’s phosphate-buffered saline
DPBST: Dulbecco’s phosphate-buffered saline + 0.1% Triton X-100
EGTA: Ethylene-bis(oxyethylenenitrilo)tetraacetic acid
EOFAD: Early onset familial Alzheimer’s disease
ESC: Embryonic stem cells
FACS: Fluorescence-activated cell sorting
GAPDH: Glyceraldehyde-3-phosphate dehydrogenase
GFP: Green fluorescent protein
HDR: Homology directed repair
HFIP: 1,1,1,3,3,3-hexafluoro-2-propanol
HRP: horseradish peroxidase
IPSCs: Induced pluripotent stem cells
LDH: Lactate dehydrogenase
MAP2: Microtubule-associated protein 2
MGE: Medial ganglionic eminences
NEBs: Neuronal Embryoid Bodies
NGF: Nerve growth factor
NLRP2: NLR family pyrin domain containing 2
NPC: Neural progenitor cells
PFA: Paraformaldehyde
PSEN: Presenilin
RNA: Ribonucleic acid
Rock: Rho-associated, coiled-coil containing protein kinase
RT: Reverse Transcriptase
RT-qPCR: Real-time quantitative polymerase chain reaction
SAG: Smoothened agonist
SDS-PAGE: Sodium dodecyl sulfate polyacrylamide gel electrophoresis
SEM: Standard error of the mean
sgRNA: Single guide RNA
Shh: Sonic hedgehog
SNP: Single nucleotide polimorfism
ssODN: Single stranded oligonucleotides
TBST: Tris-buffered saline + 0.1% Tween
VACht: Vesicular acetylcholine transporter
WT: Wild type
