# Supplementary material for: CRISPR/Cas9-Correctable mutation-related molecular and physiological phenotypes in iPSC-derived Alzheimer’s PSEN2N141I neurons
Source: Acta Neuropathol Commun. 2017 Oct 27;5:77. doi: 10.1186/s40478-017-0475-z (PMC5660456; doi:10.1186/s40478-017-0475-z)
Supplement: Supplementary file 1 — Antibodies, Species, Titers, and Vendors Used in this Study. (DOC 31 kb) [file 40478_2017_475_MOESM1_ESM.doc]

Supplementary Table of Antibodies, Species, Titers, and Vendors Used in this Study

| **Antibody** | **Host** | **Dilution** | **Company** |
| --- | --- | --- | --- |
| BF1 | Rabbit | 1:500 | Abcam |
| ChAT | Rabbit | 1:500 | Millipore |
| MAP2 | Chicken | 1:2000 | Abcam |
| Nestin | Mouse | 1:300 | Millipore |
| Nkx2.1 | Rabbit | 1:250 | Abcam |
| Sox2 | Rabbit | 1:250 | Millipore |
| TrkA | Rabbit | 1:100 | BD |
| Tuj1 | Mouse | 1:1000 | Sigma |
| VAChT | Rabbit | 1:1000 | Sigma |
