# Supplementary figures and images for: CRISPR/Cas9-Correctable mutation-related molecular and physiological phenotypes in iPSC-derived Alzheimer’s PSEN2N141I neurons
Source: Acta Neuropathol Commun. 2017 Oct 27;5:77. doi: 10.1186/s40478-017-0475-z (PMC5660456; doi:10.1186/s40478-017-0475-z)

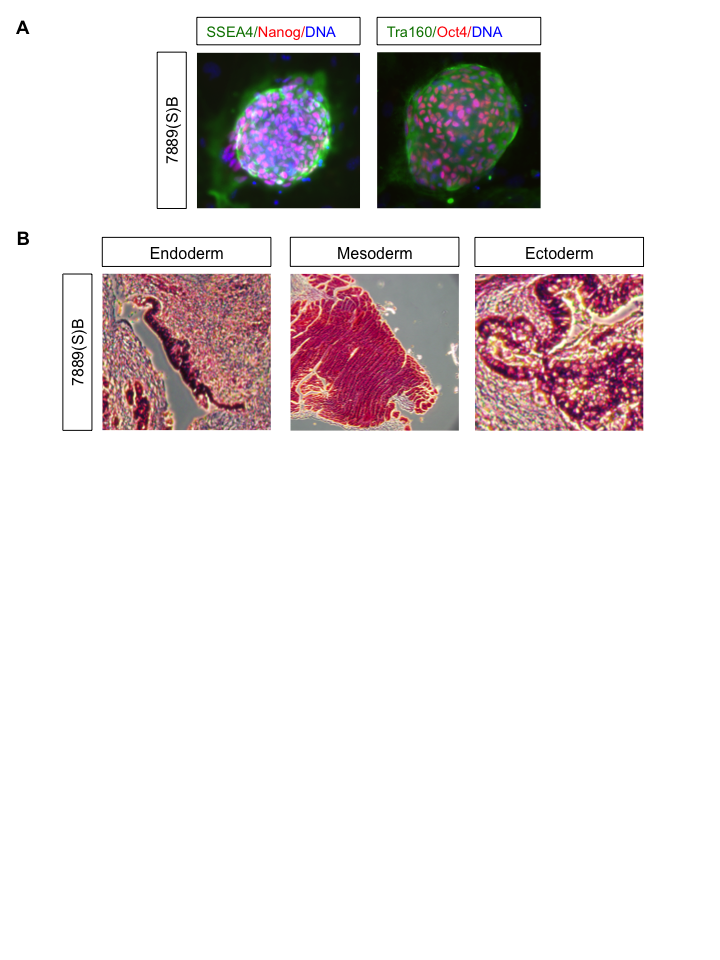

Supplement: Supplementary file 2 — Quality control of iPSC lines. (A) Immunofluorescence shows expression of pluripotency markers SSEA4, Nanog, Tra160 and in 7889(S)B iPSC line. (B) Three germ layers from teratomas generated by 7889(S)B iPSC line. (TIFF 2702 kb) [file 40478_2017_475_MOESM2_ESM.tif]

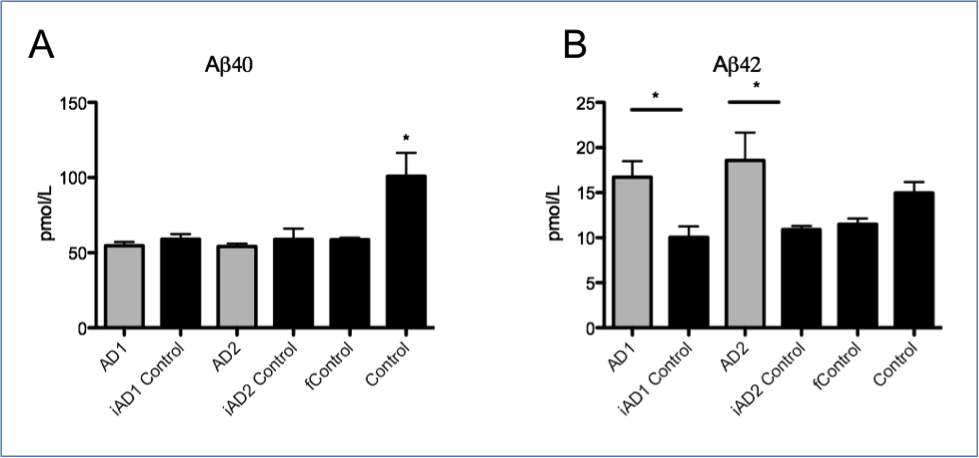

Supplement: Supplementary file 3 — Amyloid β levels in mature BFCNs. (A) Levels of Aβ40 on BFCNs (DIV 34). *, P < .01 vs. other lines in study according to One-Way ANOVA Bonferroni Post-hoc test. (B) Levels of Aβ42 on BFCNs (DIV 34). n = 3, 3 independent experiments with technical triplicates. *, P < .01 based on Student’s T-test. (TIFF 1753 kb) [file 40478_2017_475_MOESM3_ESM.tiff]
